# An Online Psychological Program for Adolescents and Young Adults With Headaches: Iterative Design and Rapid Usability Testing

**DOI:** 10.2196/48677

**Published:** 2023-12-12

**Authors:** Anna Huguet, Sharlene Rozario, Lori Wozney, Patrick J McGrath

**Affiliations:** 1 Department of Psychology Universitat Rovira i Virgili Tarragona Spain; 2 Izaak Walton Killam Health Centre Halifax, NS Canada; 3 Mental Health and Addictions Izaak Walton Killam Health Halifax, NS Canada; 4 Department of Psychiatry Dalhousie University Halifax, NS Canada

**Keywords:** adolescents, cognitive-behavioral intervention, design process, end users, headaches, internet, usability, young adult

## Abstract

**Background:**

Headache disorders are common, debilitating health problems. Cognitive-behavioral therapy (CBT) is recommended but rarely easily available. With the use of the internet and communication technologies among youth and young adults, these individuals could be self-trained in CBT skills. There is an increasing number of internet-based interventions for headaches, but there has been little research into the usability of these interventions because evaluating usability across the intervention development life cycle is costly. We developed an internet-based CBT program, the Specialized Program for Headache Reduction (SPHERE). While developing it, we aimed to improve SPHERE through rapid usability testing cycles.

**Objective:**

This study aims to presents a rapid and affordable usability testing approach that can be performed throughout the intervention development life cycle. This paper also provides evidence of the usability of SPHERE.

**Methods:**

We used the “think aloud” usability testing method based on Krug’s approach to test user interaction within a lab setting. This was followed by a short posttest interview. We planned to test SPHERE with 3-5 participants testing the same part of the program each cycle. Both the design and development team and the research team actively participated in the usability testing process. Observers independently identified the top 3 usability issues, rated their severity, and conducted debriefing sessions to come to consensus on major issues and generate potential solutions.

**Results:**

The testing process allowed major usability issues to be identified and rectified rapidly before piloting SPHERE in a real-world context. A total of 2 cycles of testing were conducted. Of the usability issues encountered in cycles 1 and 2, a total of 68% (17/25) and 32% (12/38), respectively, were rated as major, discussed, and fixed.

**Conclusions:**

This study shows that rapid usability testing is an essential part of the design process that improves program functionality and can be easy and inexpensive to undertake.

## Introduction

### Making Easily Accessible, Supported, and Self-Management Options for Headaches Worldwide

Headaches, including tension-type and migraine, are a common health problem [1] and are among the top 5 causes of disability worldwide in individuals aged between 15 and 49 years [2], with widespread societal costs [3,4]. Medication is the predominant treatment, but it may not be effective and can have side effects [5,6]. Being a multifactorial disorder, a biopsychosocial approach is considered the most appropriate option [7], where patient education and lifestyle modification are recommended in addition to medication. Behavioral approaches, specifically cognitive behavioral therapy (CBT), biofeedback, and relaxation, are effective [8-10], but acceptance and commitment therapy and mindfulness are accumulating evidence [11,12] as recommended treatments for headaches [13].

There has been a proliferation of digital health technology programs using CBT for headaches [14]. Because of the overwhelmed health care systems and the widespread access to the internet and computing devices, these technologies could be a valuable addition to current care [15]. These programs could increase patient access [13] by delivering self-management training in CBT to a large population of patients in a timely manner at modest costs. Studies conducted on the efficacy of internet-based headache CBT (iCBT) have shown promising results for adults, but few of them have been designed and tested with youth [16-18].

### Including Rapid Usability Testing Within an Iterative Design Process

Usability testing is essential when creating eHealth apps [19]. It helps app developers learn more about how people interact with different app features, discover errors, and find areas for optimization to improve the app’s user experience.

While eHealth apps are rapidly proliferating, published usability testing has been decreasing [20]. Usability testing is the least used evaluation method in clinical technologies [21], including headaches [14,22]. Fewer published usability evaluations may be related to apps being developed in the business sector rather than academia [23,24]. Developers often have limited resources and time pressures, and gathering usability data effectively and reporting results in literature is costly and time-consuming [25].

There is a need to build a knowledge base around how to rapidly and regularly deploy cost-efficient usability testing while developing digital health apps. Nielsen [26] was one of the first to advocate “discount usability testing” to facilitate iterative design and accelerate the improvement of user experience practices. Since then, new methods and guidance on rapid usability testing have been proposed [27,28], including (1) the use of qualitative methods such as the “think aloud” technique to pinpoint the most problematic issues that need to be addressed [20]; (2) recruiting loosely, if necessary, since most of the problems can be uncovered by testing with anyone, not necessarily the intended end users; (3) running short rounds of testing frequently; (4) not over recruiting as more participants per round may be unnecessary effort, and a waste of resources; (5) testing techniques where observers watch end users completing tasks from another room; and (6) combining multiple methods for detecting problems as that is more effective than any one approach [29]. Such testing can be completed in a single day, accelerate implementing changes to the program quickly, and move the intervention closer to large-scale testing.

### Our Efforts Toward Making CBT More Accessible and Incorporating Usability Testing Into Development Cycles

To make treatment for headaches more accessible, we developed an iCBT program called Specialized Program for Headache Reduction (SPHERE) for individuals aged between 14 and 28 years old with recurrent headaches. SPHERE included (1) a self-paced program aimed to educate users about their headaches and teach pain coping skills, (2) an electronic diary to track headaches and generate reports to improve understanding, and (3) an online community to facilitate the exchange of knowledge and social support.

SPHERE was created using an iterative, participatory process. As part of the initial design process for SPHERE, we performed focus groups with potential users to determine what our target population wanted, needed, and liked to be included to ensure the program could support their needs [30]. Once we defined the SPHERE structure and main content, we started development and aimed to improve its usability while it was being built by discovering major problems and fixing them before testing in the real world. We had time and resource constraints, so we used the “think aloud” approach inspired by the methodology proposed by Krug [31] in his book *Rocket Surgery Made Easy: the Do-It-Yourself Guide to Finding and Fixing Usability Problems*, in combination with a supplementary brief semistructured interview to uncover major problems. The program was tested early in the development process with users who were not representative users and later on with representative users. The focus of this manuscript is the results derived from the last testing rounds with representative users and a blueprint to illustrate a simple and fast approach that encompasses 2 usability evaluation techniques to create SPHERE.

This approach provides a resource-friendly usability methodology evaluation and helps eHealth stakeholders develop digital health care tools in clinical practice (eg, digital health tools for the NHS [National Health Service] should meet the standards required by the NHS Digital Technology Assessment Criteria, which includes research evidence of usability testing [32]).

## Methods

### Creating a Functional Prototype

A “paper” prototype version of SPHERE was first developed. SPHERE is made up of 3 areas: “learn,” “track,” and “discuss” (the paper prototype is shown in Multimedia Appendix 1).

The “learn” area contains 30 educational topics focused on headache conditions, effective treatment options, and skills and techniques they can use to reduce their headaches. Illustrations, videos, demonstrative animations, quizzes, tasks, and interactive weekly practices support engagement and learning.

The “track” area provides a web-based version of the myWireless Headache Intervention headache diary app [33], accessible and optimized for smartphones, tablets, and personal computers. The “diary” tracks headache details (eg, start and end times, intensity, and pain location) and records daily information on sleep hours and quality, mood, and exposure to potential triggers, strategies, medication use, and how headaches affected their day through standardized measures, the Migraine Disability Assessment (MIDAS) [34] or the Pediatric Migraine Disability Assessment (PedMIDAS) [35]. The “reports” are provided in 2 formats: a daily timeline showing all events entered into the diary as well as detailed graphical reports.

The “discuss” area is a discussion forum moderated by a team member. The aim of the forum is to promote learning and provide a positive community of people working through their problems together to improve collective outcomes.

**Figure 1:**
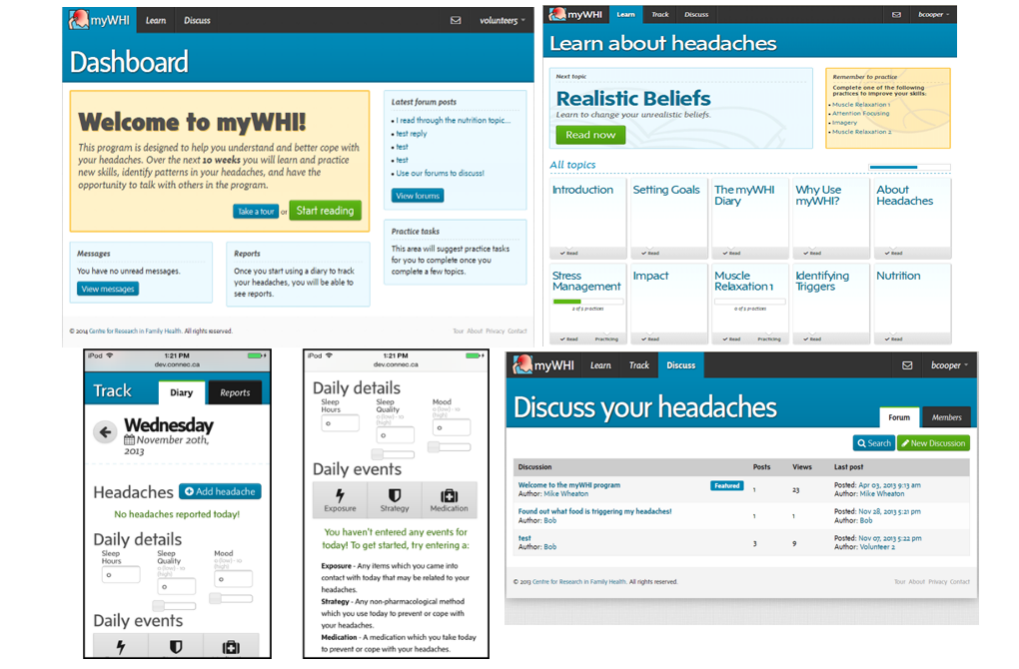
Functional prototype of parts of Specialized Program for Headache Reduction (SPHERE) at the end of the study: dashboard (screenshot on the top left), learn topic blocks (screenshot on the top right), track on a smartphone (screenshot on the bottom left), and discuss (screenshot on the bottom right).

The initial paper prototype, as well as a more detailed, yet minimally functional, mock-up, was informally evaluated with volunteers and colleagues under the assumption that almost anybody would find major usability issues [31]. We asked them to look at the paper prototype and the minimal functional mock-up and try to figure out what they were or what they would expect to see when they clicked on “here.” Using this feedback, the final step was to create a functional prototype where software developers programed the major features (eg, separate page for learn, track, and discuss*)* that could be tested through more formal usability evaluations (Figure 1).

### Procedure

An initial formal usability testing of SPHERE’s individual parts (ie, dashboard, learn, track, discuss, and content areas) was conducted with end users. After fixes were made to the major usability issues identified in cycle 1, we planned additional cycles. Most of SPHERE, including dashboard, learn, and discuss areas, were tested on a computer. The track area was tested with a smartphone, and reports were made on both a computer and a smartphone. We used these devices because the focus group study indicated what devices they would use for these parts of SPHERE [20]. Treatment content was tested on 2 randomly selected topics. Participants were asked to report on the writing style and how useful, understandable, or interesting the topics were. One other topic was assessed on paper to assess content alone without considering the effects that website features would have on displaying content.

In each cycle, a trained usability facilitator sat in the room with the participant before starting the session, confirmed consent, and administered a brief study prequestionnaire. Observers (3 members of the research team and 2 website developers) sat in another room, where a computer displayed the participant’s screen with mouse clicks and movement highlighted. Observers could hear all the audio. A scenario was read aloud to the participant (typically in the form of “imagine that you are... ...and you want to...”) and asked to complete several tasks. The facilitator encouraged participants to “think aloud” by verbalizing their internal dialogue, providing insight to understand if and why a problem may have been encountered. The facilitator only provided help if the participant was unable to continue after more than a few minutes. Once all the scenarios and tasks related to one part of SPHERE were completed and before moving to a new part of SPHERE, as well as at the end of the test, several open-ended questions through a brief interview were asked to better understand their reactions to the program as well as to capture overall impressions and additional suggestions. Krug [31] recommended only spending 5 minutes asking follow-up questions at the end of the test to help the team understand what they observed. We decided to formulate some follow-up questions after testing one part of SPHERE and before moving to another one (eg, moving from the track area to the discuss area), so that the tested part would be fresh in participants’ minds as well as engaging participants at the end of the “think aloud” process with a brief interview to get a deeper understanding of their impressions of the program. Before starting this brief interview, the facilitator briefly met with the other team members to ask whether any further scenarios or questions were needed. At the end of each usability session, the observers and the facilitator, who may have been taking notes during and after tasks, independently identified the three most important usability issues observed in each session, and the lists were compiled.

It was not possible due to participants’ time commitments to do one session after the other, as Krug [31] recommends. Instead, usability cycle sessions were scheduled within a 7- to 14-day period, depending on the availability of the participant. At the end of each cycle, the facilitator and observers participated in a debriefing session where they came to a consensus and allocated a severity grade to each problem following the grading criteria described in Table 1. Usability issues that were rated as major were prioritized for fixing, and developers implemented changes immediately afterwards.

**Table 1:** Severity ratings of usability issues.

Criteria	Definition	When does a usability issue need to be addressed? When it was rated as a major usability issue, defined as…
Frequency	Number of participants that encountered the issue	50% or more of participants encountered the same issue
Impact	To what extent the usability issue prevents task completion	The issue had a high impact on the overall user experience (ie, created major barriers to performing common tasks)
Persistence	Ability of the user to resolve the issue independently	The user is not able to resolve the issue independently

### Participants

We planned to have at least 3-5 participants test the same part of SPHERE per cycle; this number is based on research findings that show that the first 3 users are very likely to uncover the most significant problems. Research demonstrates having 3-5 participants is enough to identify 75% to 85% of usability issues [28,29]. Study participant inclusion criteria were (1) aged from 14 to 28 years, (2) experience 2 or more headaches each month for at least 3 months, (3) have experience using technology, (4) be fluent in English, and (5) consent to participate. Participants were not allowed to participate in more than one usability cycle.

### Materials

For the participant and facilitator, a standard desktop computer with a mouse and keyboard, a smartphone, screen recording software (ie, CamStudio [Microsoft Windows] for computers and built-in screen recorders for smartphones), screen sharing software r (ie, VNC viewer [The Founders of RealVNC]), software used to show mouse clicks, and a speakerphone for sending audio to observers were used. For the observers (located in a close-by conference room), a laptop computer with large external display for shared viewing, screen viewing software (ie, VNC viewer), and speakerphone with the microphone muted were used.

### Measures

#### Preassessment Questionnaire

Participants’ demographic information, headache characteristics, perceived skill levels, average use, and attitudes toward technology were collected before the session using an ad hoc questionnaire.

#### Usability Tasks

Multimedia Appendix 2 displays examples of usability tasks provided to the participants in the form of scenarios to make them use several parts of the website and observe how they use it.

#### Posttest Semistructured Interview

A 15-minute semistructured interview (Multimedia Appendix 2) was administered to gain more knowledge into participants’ reactions to the site (eg, whether they had noticed any feature, why they decided to take that action to complete a particular task), as well as their overall experience with the program and suggestions for improvements that might have helped them use the program easier.

### Data Analysis

Data collected through the prequestionnaires was analyzed using SPSS Statistics (version 28; IBM Corp). Descriptive analysis, including median for continuous and frequency counts for categorical variables, was calculated.

### Ethical Considerations

The study was approved by the Izaak Walton Killam Health Center Research Ethics Board (1012839). Participants were recruited online (eg, social media and classified sites) and screened for eligibility. Interested individuals were directed to a study website, which evaluated and automatically determined eligibility for the study. If eligible, individuals could proceed with online consent. Those consenting were contacted by the research team to schedule a time to participate in the study. At the end of each session, a CAD 10 (US $7.38) gift certificate honorarium was given to participants.

## Results

### Cycle 1

#### Participant Characteristics

A total of 4 female and 2 male participants with a mean age of 26.17 (SD 1.60; range 24-28) years participated. Types of headaches reported by participants were migraine (n=1), tension-type (n=1), and a mix of migraine and tension type (n=2), with 2 being unsure of headache type. Almost all the participants (5/6, 83%) reported having positive attitudes toward the internet and communication technologies. All participants reported using the internet, computer, and smartphone every day and having high skills using them. Lower levels of use were reported with regard to the tablet.

#### Program Evaluation

Each part of the SPHERE, including learn, reports under track, discuss, and treatment content for 2 randomly selected topics, was evaluated on a computer by 3 participants, except for the dashboard, which was evaluated separately on a computer by 6 participants, and the track area, which was evaluated on a smartphone by 5 participants.

#### Major Usability Problems and Solutions

A total of 68% (17/25) unique usability issues identified as the top 3 usability problems were rated as major. Below, we summarize the major usability issues and the changes implemented. Minor issues (eg, changing colors of directional arrows) were addressed quickly by the developers and are not discussed in this paper.

##### Dashboard (Website Home Page)

The purpose of the program was not clear, and the dashboard was not identified as the home page.

- Solution: A “Welcome to SPHERE” panel was added. The panel also included a “take a tour” button, which covered primary navigation and a high-level overview.

##### Learn

It was difficult for participants to identify their progress in the program. Most navigated to the track tab, mistaking the “tracking” of headaches with progress through the program.

- Solution: The track tab was hidden until the user’s completed the fourth topic, which introduces the diary.

**Figure 2:**
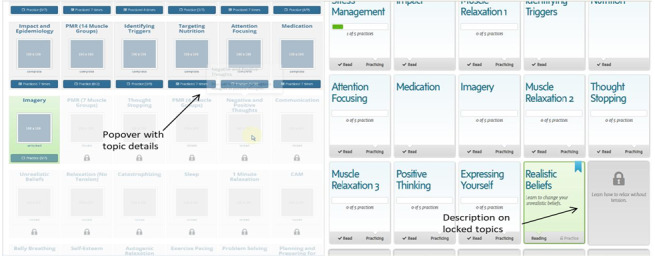
Learn topic blocks tested in cycle 1 (screenshot on the left side) and learn topic blocks after cycle 2 changes (screenshot on the right side).

It was difficult for participants to see topic descriptions and for them to identify progress within a topic (left screenshot in Figure 2).

- Solutions: (1) A larger lock icon as well as functionality were added. When participants hovered over a window, descriptions in a larger font size would pop up. (2) A progress bar for every topic was added (eg, having read 2 of 5 pages would result in a 40% progress bar; right screenshot in Figure 2).

The purpose of collapsible panels within topics was misinterpreted. Participants thought that their purpose was to shorten page length when their purpose was to present supplemental and optional information.

- Solution: An “optional” label was added along with a brief explanation in the first encounter of the participants with the panels.

Participants were unsure of how to navigate to a topic’s practice page.

- Solutions: (1) Informational text that explained the purpose of the practice page and where to find it was added. (2) The primary action of clicking on a topic widget was changed so that it would take users directly to the practice page for completed topics. (3) An arrow pointing to either the word “read” or “practicing” on each topic widget was created to prompt users to take action based on their progress (right screenshot in Figure 2).

##### Reports (on a Computer)

It was difficult for participants to pull up a report of data for a requested specific time period (eg, “the last 2 weeks” from a date).

**Figure 3:**
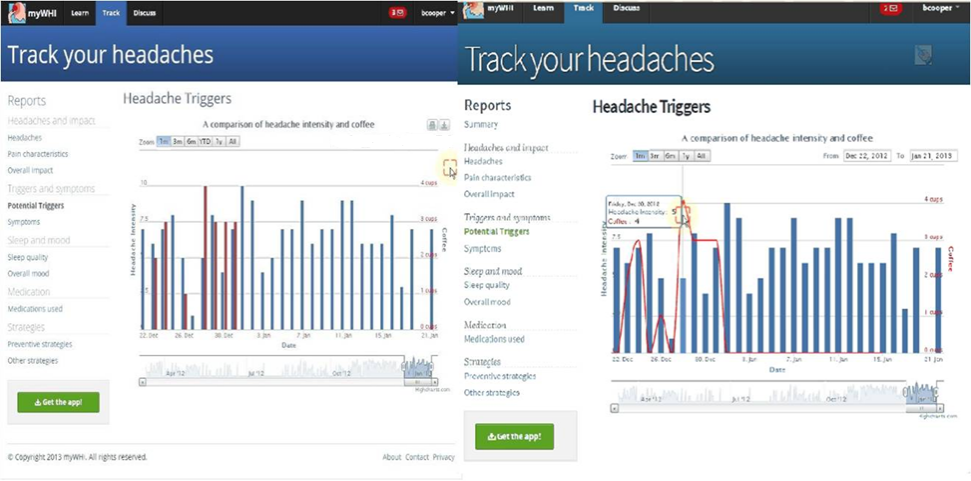
A report graph tested in cycle 1 displays headache (intensity) and coffee as potential triggers, represented as blue and red graphs (screenshot on the left side). Report graph displaying headache (intensity) as a blue bar and a potential trigger as a red dot for a scatter plot tested in cycle 2 (screenshot on the right side).

- Solution: Functionality was added to select a start and end date from a calendar (right screenshot in Figure 3).

It was difficult to interpret the trigger bar graph report (right side in Figure 3).

- Solution: Triggers were represented as a scatter plot to differentiate trigger data from headaches (right screenshot in Figure 3).

##### Track (on a Smartphone)

Buttons available for tracking daily events (eg, factors or medication) were not intuitive.

- Solution: Short instructional texts for each button and how they could be used to fill them out were added (left bottom screenshot under “daily events” in Figure 1).

The comments section, created to add limited additional details about their day not captured elsewhere, would be used for a different purpose (ie, they would use it to record what may have potentially triggered, and the diary already includes an item to record potential triggers along with its graphical report).

- Solution: The comments button was removed from individual events, and a “notes” section was created at the end of a daily diary page.

It was difficult to view and interact with reports on a smartphone, especially reports for large time periods (eg, 6 months).

- Solution: Users are encouraged in topic 4 that explains tracking to view the reports on a computer screen for optimal viewing.

Graphical and text-based reports were not understood.

- Solution: Short descriptions for each report were added to topic 4.

##### Program Content in Learn

Seeing tasks and quizzes throughout the content was confusing.

- Solution: A small explanation was added at the first activity or task to prime users about the intentionality of these features.

Readability, comprehension, and interest were suggested to be improved with the inclusion of illustrations, animations, or videos.

- Solution: Test illustrations for 2 topics were created by 4 different illustrators. We asked volunteers and colleagues to evaluate these illustrations. The highest-ranked illustrator was selected to create content illustrations for the entire SPHERE content catalog.

### Cycle 2

#### Participant Characteristics

A total of 6 female and 1 male participants with a mean age of 20.57 (SD3.55; range 14-25) years participated. All participants reported being very knowledgeable about the use of the internet, computers, and smartphones.

#### Program Evaluation

Tasks were assigned so that 3 participants read on a computer and commented on the content of a topic; 4 participants viewed the website in its entirety, including the topic’s content; and 3 participants used a smartphone to view the track area.

#### Major Usability Issues and Suggestions

A total of 12 (32%) out of 38 unique usability issues were identified and rated as major. The majority of the issues identified in cycle 1 were not identified in cycle 2 and assumed to be resolved at least until further testing. Below, we summarize the major issues.

##### Learn

It was unclear why participants have been automatically directed to the practice component when returning to a topic.

- Solution: A hovering checkmark next to the words “read” and “practice” was added to show what was or was not complete for a topic (right screenshot in Figure 2, topic widgets).

##### Track (Tested on a Computer)

Difficulties using the scroll tool for the report graph to show data over a time period were still observed. In cycle 1, this tool was hardly ever used (right screenshot in Figure 3).

- Solution: Taking into account that this tool could not be very commonly used, a new toggle button was added to allow users to choose a different time period (eg, 1 week) with the push of a button and give users options to customize different period times.

It was unclear what the buttons next to the “zoom” label, which adjust the date range of the report, would do (right screenshot in Figure 3).

- Solutions: The “zoom” word was replaced by the “scale” word.

##### Discuss

The identification of relevant discussions was foreseen as a challenge, and forum discussions were suggested to be organized by categories based on SPHERE topics.

No changes were made at that stage. However, we planned to add categories in future iterations of SPHERE if we saw enough discussions that could be meaningfully grouped.

The term “sticky post” that was used to label those posts created by the SPHERE team that users could not reply to was found unclear.

- Solution: The term “featured” was used instead (bottom right screenshot in Figure 1).

##### Track (Tested on a Smartphone)

It was not understood how to track potential triggers through the diary. SPHERE users are asked to identify up to 5 factors they want to track consistently to determine if those could be headache triggers and keep track of these daily, regardless of whether they had a headache or not. However, participants would only enter factor data on headache days or track everything they were exposed to. Both approaches are problematic because they can (1) distort the program’s ability to build associations between triggers and headaches and (2) increase participant burden.

- Solutions: No improvements were made in how potential triggers were tracked. Instead, a justification of the reasons for tracking every day was added.

The level of understanding of reports was still poor.

As reports were based on mock user data and participants had not reviewed key program information, it was difficult to determine if the cause was due to how the report was presented or a lack of meaningful connection to the data. No changes were made.

Sliders and buttons were too small and generated errors.

- Solution: The buttons and sliders were increased in size.

## Discussion

### Overview

This rapid usability study was conducted to improve the SPHERE program, designed for frequent headache sufferers. After 2 cycles of usability testing involving 6-7 participants in each, we were able to identify and rapidly address major usability issues with minimal development efforts, as confirmed by the improved results in a second cycle of testing; fewer major usability issues as well as a lower percentage of major issues were identified in cycle 2 when compared with the number and percentage of issues identified in cycle 1.

The main lessons learned by the team were that it was important when users sign in to SPHERE to immediately and briefly explain what the entire program is about rather than relying on participants discovering it through use of the program, because that is consistent with standards [36]. Second, it was beneficial to provide parts of the program only when they needed them. SPHERE was initially designed to show users all its parts from the beginning, but the results of the usability study suggested familiarity with the simplest system should happen first followed by introducing users to more complex aspects of the program (eg, diary) after they had basic system knowledge. Finally, results made it clear that more attention was needed to test alternative paths through the app because end users had been observed taking diverse approaches (eg, when pulling a report of data for a specific time period or when navigating to several sections of the site, the main path that was designed to complete these tasks was not the most commonly chosen by participants).

Following Krug’s [31] recommendation, we decided to first implement minimal changes involving the least effort possible to fix major problems with the user interface. When we did, we found that many major usability issues were resolved. However, there were still major issues uncovered in the first cycle that were not satisfactorily resolved after conducting the second cycle of testing (ie, reports were still not understood). The lack of understanding of reports identified in this study could be only a problem related to a mismatch between the program and the context in which it was tested. In the lab, for the purpose of testing SPHERE through the “think aloud” procedure, several dummy users were created, and participants, who were exposed to the whole program at once and not given the opportunity to learn how to track and learn, were asked to interpret the reports of these dummy users. Therefore, in the laboratory, we were not able to provide participants with actual situational context to complete the tasks properly. For this reason, as Hertzum [37] argues in his essay, usability should be evaluated early, but also later, when the system is sufficiently functional and robust to be tested in the field. Consistently, in an attempt to be efficient and taking into account that the percentage of major issues in the second cycle had decreased considerably, we decided instead of conducting a new round of usability testing in the laboratory to get the program ready to be used in a real-world context for a restricted period of time. This new evaluation would give us an opportunity to explore whether the issues identified in cycle 2 had been successfully fixed and a new opportunity to uncover new major usability issues. Then SPHERE would be refined and studied in a randomized controlled trial to determine its overall effectiveness in improving headaches.

The inclusion of website developers as observers in our testing protocol was a recommended approach [38]. This helped us to explore, based on a few actual users, if chosen design features and navigational tools were interpreted in the same way or differently from what they expected and make changes to the program according to user feedback. Moreover, having the SPHERE designers and developers observe the session allowed them to catch other issues that may not be apparent to other observers (eg, links rerouting participants to the wrong page or not rerouting them, broken links, or bugs in the system).

Leveraging multiple sources of data (ie, direct observations of user-system interaction, verbal comments given by the user during the “think aloud” sessions, and data from interviews) is a recommended practice [39,40] and allows us to gain a more comprehensive understanding of the user’s experience when interacting with SPHERE. For instance, posttesting interview data not only corroborated issues participants had encountered during the “think aloud” technique but also allowed for solution-generation in more detail. For instance, by using interview methods, participants gave us ideas about how to improve difficult parts of the system (eg, confusing words or graphs).

Our findings contribute to necessary discussions on how to improve iterative and early usability methodologies so eHealth evidence-based apps can be developed more efficiently. It is very important to improve the usability of self-management programs for headaches because poor usability design can contribute to the low adherence and high attrition rates observed in trials of self-management programs for headaches [17], and consequently, affect treatment outcomes.

### Study Limitations

This study presents some limitations. First, although we implemented 2 usability methods often used (ie, “think aloud” technique and interviews), the way these methods were implemented in this study has not been empirically validated. Second, the limited number of youth recruited (only 1 in the 14-16 years age bracket) may limit the representativeness of this group in the study. Third, we did not transcribe and perform qualitative analysis of video recordings of usability test sessions and posttask interviews, which is a common practice in more academic usability testing [41]. We followed Krug’s [31] recommendation, and we did not perform data analysis. Instead, we relied on our session observation notes and memories. It is possible that this less expensive, more rapid approach led to incomplete or biased observer ratings. However, to reduce bias, observers were trained beforehand, and we ensured several observers in each test session to reduce the undue influence of any one observer. Lastly, the design of the testing process may have altered how participants interacted with SPHERE. Participants were asked to pretend that they were using the program as both a completely new and experienced user (eg, data were prepopulated into the program to show visualizations for timelines and graphical reports). It may have been confusing for users to provide feedback on what they were told was expected to happen versus what they themselves were discovering as they used the program. However, we were still able to identify many major usability issues, which is the most critical focus of usability testing [31].

### Conclusions

In summary, through this rapid method of usability testing that incorporated “think aloud” technique and interviews focused on identifying major problems, we were able to make considerable enhancements to an early prototype of SPHERE, and a subsequent cycle provided some evidence that we introduced no other major issues once these changes were made. The findings will be of interest to those developing similar interventions or trying to learn more about how users interact with web-based iCBT programs. The methods described could be incorporated by others in the design of related eHealth apps.

## Appendix

Multimedia Appendix 1 Paper prototype of the Specialized Program for Headache Reduction (SPHERE): dashboard (home page), learn area (topics), track area (diary and reports), and discuss area (community).

Multimedia Appendix 2 Examples of scenarios and questions asked when testing Specialized Program for Headache Reduction (SPHERE).

## Abbreviations

CBT: cognitive behavioral therapy
iCBT: internet-based headache CBT
MIDAS: Migraine Disability Assessment
NHS: National Health Service
PedMIDAS: Pediatric Migraine Disability Assessment
SPHERE: Specialized Program for Headache Reduction
