# Supplementary material for: An Online Psychological Program for Adolescents and Young Adults With Headaches: Iterative Design and Rapid Usability Testing
Source: JMIR Hum Factors. 2023 Dec 12;10:e48677. doi: 10.2196/48677 (PMC10751633; doi:10.2196/48677)

Paper prototype of the SPHERE Dashboard (Homepage), Learn page (Topics), diary reports and Discuss (Community).


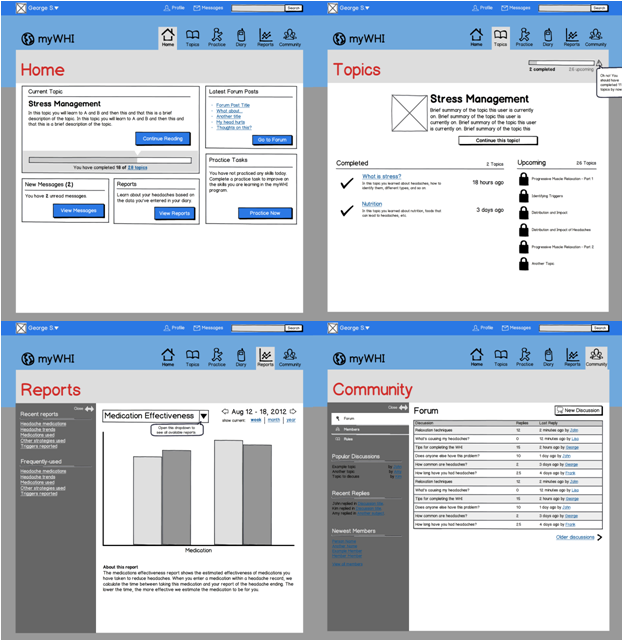

Supplement: Multimedia Appendix 1 [file humanfactors_v10i1e48677_app1.docx]
