# Supplementary material for: An Online Psychological Program for Adolescents and Young Adults With Headaches: Iterative Design and Rapid Usability Testing
Source: JMIR Hum Factors. 2023 Dec 12;10:e48677. doi: 10.2196/48677 (PMC10751633; doi:10.2196/48677)
Supplement: Multimedia Appendix 2 [file humanfactors_v10i1e48677_app2.docx]

Examples of scenarios and questions asked when testing SPHERE.

| **Scenario and tasks** | **Questions made after one part of SPHERE was tested or during the post-test interview** |
| --- | --- |
| **Dashboard** |  |
| Imagine that you are enrolled as a participant in SPHERE program. You have just signed in and are presented with this page. Can you explain me what you make of it? What options are available to your from this page? | - What impression do you get of the website? |
| **Learn** |  |
| Imagine that you are part way through completing the SPHERE program. You would like to see your progress to review what you have learned and which topics are coming up next. Please find a page where you can view the details of your progress through the program. |  |
| **Learn (within a Topic)** |  |
| Now you would like to continue reading the program by completing the next available topic. As you complete the topic, please speak aloud whenever you are navigating, unsure of what is expected, or have any other thoughts. There is no need to read aloud the content itself. | - Is the navigation clear? Did you know where you were within a topic and where to go? - How do you find the visual presentation of the topic? Is the text easy to read? Can you clearly see the structure of the content? |
| **Practice** |  |
| You would like to improve your skills with imagery. Please practice this skill now. | - Did you encounter any problems while you were practicing this skill? |
| **Track** |  |
| Now you would like to see a graphical representation of your headaches over the past month. Please open the page that shows this information. What is this report/graph showing you?  Can you show me headaches you had in the past month? 3 months? Week? | - How easy was it for you to understand each report? - For you, how useful are these reports? |
| **Discuss** |  |
| Another feature of the SPHERE is that it allows you to communicate with others who are using the program. Can you please find the page where you can see what other users are doing or talking about. Can you enter a discussion from here? How? There are other users of the SPHERE, can you please identify them? | Was the information presented here enough, were you confused by anything, or did you feel that there was more information required to make you understand this section better? |
| **Printed Content** |  |
| Now imagine that you are completing the SPHERE program and have reached the topic on stress management. I’m going to give you the topic; please read it as you typically would. I can’t discuss it with you until you are done, so if you find anything confusing or have other suggestions, please just make a mark there and we will discuss it after. | - How useful did you find the information? - Were the tasks included within the topic easy to understand and complete? |
